# Obesity in achondroplasia patients: from evidence to medical monitoring

**DOI:** 10.1186/s13023-019-1247-6

**Published:** 2019-11-14

**Authors:** Celine Saint-Laurent, Laura Garde-Etayo, Elvire Gouze

**Affiliations:** 1 Université Côte d’Azur, CNRS, Inserm, iBV, Nice, France; 2 L. NUNTIA Gabinete de Orientación Nutricional SLL, Pamplona, Spain; 3 iBV, institute de Biologie Valrose, Univ. Cote d’Azur, Batiment Sciences Naturelles, UFR Sciences; Parc Valrose, 28 avenue Valrose, 06108 Nice Cedex 2, France

**Keywords:** Achondroplasia, FGFR3, Obesity, Children, Recommendations, Nutrition

## Abstract

Achondroplasia is a rare genetic disease representing the most common form of short-limb dwarfism. It is characterized by bone growth abnormalities that are well characterized and by a strong predisposition to abdominal obesity for which causes are unknown. Despite having aroused interest at the end of the 20 h century, there are still only very little data available on this aspect of the pathology. Today, interest is rising again, and some studies are now proposing mechanistic hypotheses and guidance for patient management. These data confirm that obesity is a major health problem in achondroplasia necessitating an early yet complex clinical management. Anticipatory care should be directed at identifying children who are at high risk to develop obesity and intervening to prevent the metabolic complications in adults. In this review, we are regrouping available data characterizing obesity in achondroplasia and we are identifying the current tools used to monitor obesity in these patients.

## Introduction

Achondroplasia is a rare genetic disease representing the most common form of short-limb dwarfism affecting approximately 250,000 people worldwide [1]. Mechanistically, achondroplasia is an autosomal dominant disease caused by a mutation in the fibroblast growth factor receptor 3 (FGFR3) gene [2] that corresponds to a Gly380Arg substitution in 90% of the cases [3–5]. This mutation induces the hyperactivation of the Ras/Mitogen-Activated Protein Kinase (MAPK) signaling pathway inhibiting the proliferation and differentiation of chondrocytes [6–8]. By opposition, hyperactivation of this pathway plays a positive role in adipocyte differentiation. Achondroplasia is characterized by bone growth abnormalities whose mechanisms are well-known and by a strong predisposition to abdominal obesity for which causes are not completely understood [1, 9–11].

Obesity has nevertheless been recognized for decades as a major health problem in achondroplasia and, although this has not been formally demonstrated in meta-analysis studies, it is believed to aggravate complications such as lumbar spinal stenosis, joint pain or sleep apnea [12]. Some clinical observations showed that, in the context of achondroplasia, this atypical visceral obesity development is not associated with a diabetic profile but rather with low insulin and glucose levels [11, 13, 14].

The need for a management of obesity nevertheless became obvious and in 2008, Julie Hoover-Fong proposed a new sex- and age-specific body mass index (BMI) curves in achondroplasia children in the US [15]. Several growth curve references have now been established in the different continents [16, 17]. However, the medical monitoring of patient is complex because there are no established standards available to evaluate obesity in achondroplasia patients and in particular in children making difficult to perform an effective clinical management. The goal of this revue is to propose a monitoring protocol in achondroplasia in relation with the nutritional status assessment. The early detection of obesity will help prevent and eventually treat it by proposing adapted dietary recommendations.

### Specific characteristics of obesity and metabolic status in achondroplasia

#### Atypical and early development of obesity

In achondroplasia, the development of obesity is not typical. As early as at the end of second World War, Wade H. Brown and Louise Pearce described a spontaneous rabbit model of achondroplasia with a recessive phenotype and early mortality in the first days after birth [18]. They noticed “a curious appearance due to conspicuous folds of loose, redundant skin and a very large protuberant abdomen”. In 1990, Owen et al. observed the preferential accumulation of abdominal adipose tissue in adults with achondroplasia [13]. In a retrospective study performed in children with achondroplasia, we have identified that the development of an abdominal obesity occurs very early in childhood and is exacerbated during adolescence [11]. This preferential abdominal development was confirmed in achondroplasia mice where, in spite of a similar increase in total fat mass following a high fat diet, the adipose tissue repartition was different between wildtype and achondroplasia mice, with an important increase in the development of epididymal adipose tissue in mice with achondroplasia while control mice preferentially developed an increase in subcutaneous adipose tissue [11].

#### Glucose metabolism modifications

Interestingly, in the context of achondroplasia, the development of a visceral obesity was not associated with the development of a diabetic profile and patients even had a tendency to lower fasting glycemia growing up [11]. Very similarly, achondroplasia mice developed an android obesity that was not associated with a diabetic profile, with fasting glycemia and lipidemia levels that were below normal ranges [11]. Our study demonstrated alterations in glucose metabolism triggered in part by abnormal pancreatic function and development. Moreover, indirect calorimetry showed an alteration in the use of glucose. Indeed, the mice carrying the mutation draw their energy preferentially from the oxidation of lipids, while the mice of the control group draw it from the oxidation of carbohydrates [11]. While surprising, these results have been suggesting almost five decades ago. In 1971, Thomas H. Shepard and his team used a spontaneous rabbit model of achondroplasia to better characterize the cartilage from a biochemical and physiological point of view, investigating glucose metabolism in cartilage explants [19]. They showed a defective glucose metabolism with no alterations in carbon dioxide and lactate production. Based on these preliminary results, in 1972, Plato J. Collipp et al. proposed to study the carbohydrate metabolism of achondroplasia patients aged from 4 months to 10 years [20]. Oral glucose tolerance tests were performed and Collipp concluded that an abnormal glucose tolerance was detected in 16 out of the 24 cases, suggesting a defect in peripheral glucose utilization. Although precautions need to be taken in interpreting these results because of the absence of an age-matched control group and the overly heterogeneity of this group of patients, they were nevertheless precursors of the observation of an alteration in glucose metabolism in patients with achondroplasia. In 2009, Alatzoglou et al. demonstrated the absence of insulin or glucose intolerance (defined as a plasma glucose concentration greater than 7.8 mmol/L 2 h after glucose bolus during oral glucose tolerance test (OGTT)) associated to low fasting insulinemia in adolescents with FGFR3 mutations [14]. However, in average size children, it was shown that, during OGTT, insulin levels reflecting insulin tolerance correlated better with the metabolic status of obese children than glucose levels [21]. Today, there is no study describing insulin levels during OGTT in achondroplasia children but, in one case of an achondroplasia child, insulin levels appear to be within normal standard range during an OGTT (unpublished data). If these results are confirmed, this observation will be consistent with Alatzoglou’s data describing normal glucose and insulin tolerance and low fasting insulinemia [14].

### Origin of obesity in achondroplasia

#### Energy balance

In the general population, the high prevalence of obesity has its origin in the interactions between genes and the lifestyle (physical activity, eating habits, sleep habit, stress, etc.). In achondroplasia however, before the obesogenic environment was installed in modern society, obesity was already described as a complication/disease common to people with this disease [10, 22]. Even today, the hypothesis that prevails between public and professional-sanitary opinion suggests that the predisposition to obesity and its high prevalence in achondroplasia people results from a problem of energy balance due to excessive caloric intake and lack of physical activity. Certainly, children with achondroplasia are limited by their psychomotor development and their physical condition [23]. In addition, the early appearance of overweight and obesity contributes to worsen a sedentary lifestyle and/or to exclude these children from the sports practiced by children their age. Moreover, it is important to take into account the permanent psychological stress that supposes to coexist with the social stigma of achondroplasia. Children with achondroplasia and obesity must face a double stigma and social exclusion, or fear of it, can also be a cause of stress, anxiety, hyperphagia, even binge eating and finally, obesity. It is true that it appears that children need to eat continuously, which significantly can contribute to an excessive caloric intake. It is necessary to better understand this phenomenon that seems of great importance, and is a reason for alarm for families, since it seems that children with achondroplasia want to eat all the time because they are always hungry. Very interestingly, this phenomenon of constant feeding has been observed in a study using a mouse model of achondroplasia. In mice, the energy expenditure and the total food intake was not modified but they have a tendency to eat constantly over a 24 h period [11]. These observations demonstrate that the phenomenon of constant eating seems to be an important factor when we considered obesity in the case of achondroplasia. It is necessary to discern if this behavior is a response to a psychological or physiological-metabolic problem, or both. Finally, scientific studies describing energy expenditure in people with achondroplasia concluded that the basal metabolism of people with achondroplasia is lower than that of people of average height of the same age and sex, but higher than expected for their height and weight [13, 24–27]. Although none of them is conclusive, different hypotheses could explain the high metabolic rate per unit of weight in people with achondroplasia at rest as well as practicing physical activity, compared to people with standard average height. Obviously, body composition and body proportions are different between people with standard height and people with achondroplasia and it is necessary to determine the relationship between body composition and resting energy expenditure (REE) in achondroplasia. On the other hand, the anthropometric differences observed in these people determine a higher stride frequency, a higher breathing rate and a higher heart rate and, therefore, a higher REE. Similar to the population of standard height, there is great interindividual variability. In a recent study, Madsen and collaborators describe the anthropometrics, diet, and REE in a group of Norwegian adult population with achondroplasia [28]. The results revealed a high frequency of central obesity and unhealthy dietary habits. Mean energy intake was low and only 10% higher than the mean REE and does not explain the high prevalence of abdominal obesity observed. Altogether, the different studies strongly suggest that the origin of the visceral obesity in achondroplasia cannot be limited to energy imbalance.

#### FGFR3 mutation

The link between the mutation responsible for achondroplasia and the metabolic complications seems difficult to highlight because no metabolic pathology has been directly associated with a mutation on the FGFR3 receptor. Indeed, the only known pathologies whose origin is a mutation on the FGFR3 receptor are chondrodysplasias, multiple myeloma and some cancers. Achondroplasia remains one of the rare cases proving a possible relationship between FGFR3 signaling and the development of metabolic alterations. However, today, there is an increasing number of evidence suggesting that the early onset and development of this central and atypical obesity may originate in the FGFR3 mutation itself [11, 13, 20]. Using a mouse model of achondroplasia, we have recently hypothesized that an alteration in the biology of mesenchymal stem cells, that are common chondrocytes and adipocytes progenitors, could partially explain the development of obesity in the context of achondroplasia [11]. Our hypothesis is in agreement with a recent study showing that mesenchymal stem cells generated from an achondroplasia patient carrying the G380R mutation exhibited significantly reduced osteogenic differentiation and enhanced adipogenic differentiation [29]. In this sense, several studies have shown that the activation of one differentiation pathway involves an inhibition of the other one [22, 30–32] confirming a permanent regulation loop between adipocytes and chondrocytes differentiation. Oxytocin whose receptor is present both in adipocytes and chondrocytes could play an important role in this regulation [30]. Very interestingly, in accordance with a previous study demonstrating that the inhibition of hypertrophic chondrocytes differentiation is mediated by Erk [33], the study in mesenchymal stem cells generated from a patient confirms that hyperactivation of this pathway could be responsible for the increased differentiation of adipocyte progenitor [18]. Moreover, it has been suggested that the Oxytocin action in adipocyte differentiation could be mediated by Erk1 / 2 phosphorylation [30] and it would appear that the Oxytocin - FGFR3 / Erk axis plays a key role in the establishment of the phenotype observed in achondroplasia.

Other evidence suggest that mutations in the FGFR3 receptor responsible for bone growth alterations could be associated with the development of obesity. A report presenting a case of hypochondroplasia associated with acanthosis nigricans describes high BMI associated with insulin resistance without diabetes development [34]. This increase in BMI was also observed in a 16 years old patient with hypochondroplasia (unpublished data). Therefore, it seems that some pathologies associate mutations in the FGFR3 receptor at the origin of a bone pathology and the development of obesity. In this sense, the FGFR3 receptor could be a common player in the complications observed. This hypothesis needs to be confirmed but they can offer new possibilities for the global treatment of achondroplasia.

### Complications linked with obesity in achondroplasia

Although this has not been formally demonstrated in meta-analysis studies, it is believed that in the context of achondroplasia, obesity could aggravate complications associated with the pathology. Indeed, the early development of obesity exacerbates joint paint, leg deformities and the potential neurologic and orthopedic complications in the lumbar spine by increasing lumbar canal stenosis [35, 36]. Several studies have linked obesity with an increased risk of sleep apnea in children and adults [37, 38]. A study conducted over 42 years showed a two-fold increased incidence of cardiovascular diseases in achondroplasia patients compared to the average size population suggesting that this could be linked to the increase of the waist-to-hip ratio [39]. Therefore, in achondroplasia patients, obesity could worsen the risk of sleep apnea and early cardiovascular mortality observed [23, 38, 40].

### Current medical management of obesity in achondroplasia patients

#### Dietary recommendations based in clinical evidences

In order to better manage obesity, it is necessary to considerer the potential nutrigenetics effect of the FGFR3 gene in the context of achondroplasia. Several molecules such as the Fat Mass and Obesity-associated protein (or alpha-ketoglutarate-dependent dioxygenase FTO), Melanocortin 4 Receptor (MC4R) or Apolipoprotein (APOE), participate in the regulation of appetite, thermogenesis, adipogenesis and can present polymorphisms allowing individualized dietary treatment according to the risk alleles carried [41]. There is little evidence available so far regarding the FGFR3 gene, therefore, recommendations for these patients should be general, since there is no optimal diet for the prevention or treatment of obesity [42].

In the context of achondroplasia, if the metabolic response to different dietary interventions is conditioned by the nutrigenetics, the question is to know what quantity and quality of macronutrients in the diet is the most appropriate. The percentage of macronutrients with respect to the total energy value of the diet may be one of the keys to dietary intervention in achondroplasia and, if this is confirmed, it would be necessary to readjust the percentages recommended of proteins, carbohydrates and fats, to their real needs. Intervention studies should be carried out to show the metabolic response after the administration of different proportions of carbohydrates, lipids and proteins in the diet. In addition to carbohydrates or fats, other nutrients or substances contained in foods with the ability to trigger or modulate the metabolic response should also be considered.

##### Carbohydrate status

As seen above, it has been observed a preference in fat oxidation over carbohydrate oxidation when the type of diet is the same for both groups (achondroplasia and control group), suggesting a conditioned metabolic response [11]. Based on this scientific observation, it could be necessary to reduce or eliminate carbohydrates in the form of free sugars (sugar, soda and energy, commercial juices and natural, sweets of all kinds, chocolate) that do not provide any nutritional benefits and could not represent an energy source in this context. The consumption of carbohydrates from fresh fruits and vegetables that are accompanied by antioxidant vitamins and protective phytochemicals should be favored. The complex carbohydrates of choice should be all those of full grain (legumes, unrefined grain cereals) and cooked *al dente*. Cereal versions devoid of their husk are not beneficial, with a higher glycemic index that favors a greater and faster increase in blood glucose. The drastic elimination of beneficial carbohydrates that also have a prebiotic effect due to their richness in dietary fiber would be unhealthy in the short, medium and long term, since it would contribute to increasing inflammatory factors and, among other things, would modify the balance of the microbiota intestinal [43].

##### Lipid status

If evidence is consolidated that lipids can be an important energy substrate of the diet of people with achondroplasia, it will be essential to promote the consumption of monounsaturated and polyunsaturated lipids with cardioprotector effects from food sources of vegetable origin (virgin oils from olive and seeds), nuts, as well as fats from blue fish. In contrast, we would recommend to reduced consumption of saturated fats (food sources of animal origin except fish) and trans or hydrogenated fats (pre-cooked foods and all most those food products that made industrially as biscuits, bakery products, pastries, fast food), whose consumption is related to a higher prevalence of inflammation, obesity and cardiovascular disease. Certain liposoluble vitamins (A, E, D), omega 3 and other substances such as resveratrol, vitamin C and folates, flavonoids, zinc and foods that contain them, could play an important role to combat the inflammatory process and oxidative stress [44–46]. In a study published in 2015, Karen L. Posey demonstrated the efficacy of resveratrol treatment applied from birth, in a mouse model with pseudochondroplasia, reducing inflammation and oxidative stress early in the disease process, compared to the control group untreated [47].

##### Psychological status

Precision dietary recommendations for the prevention and treatment of obesity and their comorbidities in achondroplasia patients must consider all personal aspects from genotype, phenotype, clinical history, physical activity, previous dietary history, socioeconomic status but also psychological status [48]. We must not forget that recommendations, actions, therapies, treatments, ..., directed at patients should contribute to improve their overall health and their quality of life (QoL). Prevention is to promote long-term health. When prevention is too late, improvement in QoL should be approached in a personalized way, setting realistic goals and offering close and frequent follow-ups. Drastic changes in the dietary pattern are not well accepted by patients, so the diet should conform to the patient’s habits and gradually modified to achieve the dietary goals that have been raised initially [49].

Therefore, dietary recommendations for people with achondroplasia should include guidelines that provide a feeling of emotional well-being and satisfaction with food. It is also necessary to pay attention and give importance to bodily sensations (feelings of hunger-satiety-satisfaction), which contribute to raising awareness and help to make food decisions with a more objective criterion [50]. The use of relaxation techniques to learn to cope with stress situations of any kind are tools that can help patients to make food decisions in favor of their well-being.

#### Bariatric surgery

Two cases of bariatric surgery have been reported in achondroplasia patients suffering from morbid obesity, highlighting the complexity of any surgery in a pathology like achondroplasia [51, 52]. Indeed, the complications associated with achondroplasia associated with those lonked with obesity make aesthesia procedures very complex, particularly with regard to respiratory disorders secondary to a deformed airway. Although two cases have been reported, bariatric surgery is totally contraindicated in addition to its own risks because it does not guarantee results in the medium and long term. Moreover, this technique only allows secondary treatment because it does not treat obesity at its source. Psychological therapy, food education, and active life, are the most effective and realistic solution to face a problem such as obesity in the context of achondroplasia.

### Assessment of nutritional status in achondroplasia patients as part of a health monitoring protocol

Obesity should be treated as a chronic disease in achondroplasia. For this reason, clinical units specialized in achondroplasia should include continuous and multidisciplinary monitoring programs where they can supervise these people adequately over time. Characterizing obesity in achondroplasia, knowing the metabolic mechanisms that act to promote its development and studying the metabolic responses to different dietary-nutritional strategies are key for its prevention and treatment. Indeed, the study of body composition and the analysis of REE in these people present many limitations and published studies are not conclusive.

#### Tools used to estimate adiposity

There are no predictive equations to estimate body fat mass in people with achondroplasia. The methods of choice for the analysis of body composition in people with achondroplasia are DEXA (dual energy X-ray absorptiometry) and magnetic resonance imaging [11, 26]. Recently, Sims and collaborators explain that, although conclusive results of whole-body or segmental body composition have not been achieved in this population group, it seems more appropriate to perform the composition of individual segments and regions to help define the clinical status of these people, instead of using whole body measurements, such as body mass index (BMI) [53]. However, in clinical practice, it is not usual to have such sophisticated equipment on hand. For this reason, anthropometric tools can still be useful in the routine monitoring of these patients.

The BMI is an height-dependent index and it can tends to overestimate body fat mass in achondroplasia patients. The BMI should be used with other complementary anthropometric measures since it does not allow quantifying fat mass or its distribution in the body. A recently published study found a strong correlation between BMI and waist circumference and describes a high prevalence of abdominal obesity that increases with age and a more sedentary lifestyle, in its group of Norwegian adults with achondroplasia [28]. However, it concluded that it is necessary to continue investigating body composition, fat distribution and its clinical implications in these people, as well as the influence that physical activity can have on these factors.

The BMI, the Rohrer index, the weight/height index, the thickness of the skin folds, the waist/hip index, the waist circumference, the fat mass and the fat-free mass are indexes, anthropometric tools and estimators of the fat mass that are available to characterized the body composition of the population with achondroplasia (Table 1). Analyzed together, they can provide information on the nutritional status and body composition of the individual, a diagnosis of the obesity situation and a proposal for adequate intervention through nutritional advice and physical activity habits. Their periodic evaluation, during the growth period can alert on situations of risk to intervene immediately and accurately [54]. Their analysis is useful for the individual follow-up of patients, but it would also be interesting to carry out a longitudinal follow-up from childhood to measure the probability of developing obesity and its comorbidities during adulthood, as well as to measure the risk of morbidity and mortality. Also, this follow-up can determine which of the anthropometric tools are most useful for the clinical management of obesity in people with achondroplasia.

**Table 1:** Advantages and limitations of tools used to characterized obesity in achondroplasia patients

	Advantages	Limitations in achondroplasia patients
Body mass Index (BMI): Weight/Height^2^ (kg/m^2^)	Easy to measure and low cost Routinely used to evaluate obesity (its use has been standardized as a tool to diagnose overweight and obesity) Standards values available in children and adults in general population *(Cole* et al.*, 2000)* Useful to epidemiological studies	Height dependent: overestimated in short stature patients *(Hecht* et al.*, 1988)* It is not a good predictor of obesity because it is not predictor of body fat and does not report on the distribution of fat (subcutaneous body fat / visceral body fat) No Standard values available in achondroplasia, only some reference curves from 0 to 16 years old *(Hoover-Fong* et al.*, 2008; Hoover-Fong* et al.*, 2016)* but nothing after 16 years old or in adults
Adipocyte rebound	Easy to measure and low cost Standards references available Early predictive to adult obesity and associated complications in general population *(Guo* et al.*, 2000; Koyama* et al.*, 2014)*	Based on the use of BMI (cf BMI limitations) No Standard values available in achondroplasia
Rohrer’s index: Weight/Height^3^ (kg/m^3^)	Easy to measure and low cost Better estimator of obesity than the BMI in children between 6 to 18 years old *(ref)* Moderated correlation with height: the best index from age 6 to 18 years in achondroplasia patients *(Hunter* et al.*, 1996)*	No standard values available in achondroplasia
Weight/Height ratio (kg/m)	Easy to measure and low cost Standards values available in general population	No Standard values available in achondroplasia, only one reference curve *(Hunter* et al.*, 1996)*
Waist circumference (cm)	Easy to measure and low cost Height independent It offers complementary information to the waist / hip ratio and both are used as predictors of cardiovascular risk Standards values available in general population	No standard values available in achondroplasia
Waist/hips ratio	Easy to measure and low cost Height independent Correlated with total fat mass in general population Used as an index of cardiovascular risk prediction due to its relationship with visceral fat in general population	No standard values available in achondroplasia
Skinfold thickness (mm)	The measurements must be made by a qualified professional and low cost Height independent Correlated with total fat mass in general population Useful to determine the subcutaneous fat mass that could correlate with orthopedic complications	No standard values available and no specific predictive models to estimate the percentage of body fat in achondroplasia They do not estimate visceral body fat, therefore, they do not serve as a tool that correlates body fat with the risk of suffering metabolic complications associated with obesity in achondroplasia patients Difficult to measure and unreliable in patients with morbid obesity
% body fat mass	Body fat mass defined obesity and is directly correlated with it Gold standard techniques (DEXA and others) are expensive and not accessible for clinical use, but specific predictive models for gender and age are available that reliably estimate body fat percentage in general population	No standard values available in achondroplasia All gold standard techniques (Dual-Energy X-ray absorptiometry technique) have important limitations as body fat estimators in achondroplasia (data on body density, body dimensions, etc.)
Androïd: gynoïd fat mass ratio (DEXA)	The most appropriated technique for correlating android obesity (estimates visceral fat independently of subcutaneous fat) with associated metabolic complications such as type II diabetes in the general population (*Aucouturier* et al.*, 2009; Samsell* et al.*, 2014; Walton* et al.*, 1995*) It can predict the development of obesity regardless of size Predictive models to estimate the percentage of body fat by measuring the skinfold can be determined in comparison to DEXA measurements.	No standard values available in achondroplasia All gold standard techniques (Dual-Energy X-ray absorptiometry technique) have important limitations as body fat estimators in achondroplasia (data on body density, body dimensions, etc.)

#### Health monitoring protocol

The impact of obesity on the overall health of people with achondroplasia is being widely discussed recently. However, obesity prevention and treatment programs that promote the long term health improvement of these patients have not been yet established. In the absence of other scientific evidences and based on the clinical experience of the treatment of obesity in people with achondroplasia, it would be necessary to propose a multidisciplinary monitoring program that integrates the tools and therapies currently available for the prevention and treatment of obesity of people of average height, adapted to the needs of this population group.

This program must be supported by two strategies: a protocol to assess the nutritional status of people with achondroplasia from birth, with the aim of preventing the development of obesity and detecting risk situations that should be treated (Table 2). And, on the other hand, a personalized action plan for each patient with the aim to work individually the nutritional aspects (dietary / nutritional recommendations, food education and nutritional coaching), psychological therapy and sports practice, more adequate in each case. In the short term, the objectives should focus on individualized patient care. In the long term, the objectives are to promote the health and QoL of these people through the prevention and/or treatment of obesity and its possible metabolic, medical and orthopedic complications. The acquisition of healthy eating habits and the practice of physical exercise since childhood should be integrated into daily routines for the promotion of long-term health.

**Table 2:** Protocol for the evaluation of the nutritional status of people with achondroplasia from birth

	Measures and data to register	Indices and results to be monitored
Anthropometric assessment (in all follow-up visit except skin folds, from 3 years old)	Weight Height and height sitting	BMI, Height/Weight, Rohrer Verify according to the reference percentile tables
	Cranial perimeter	Verify according to the reference percentile tables
	Skinfolds thicknesses (triceps, biceps, abdominal, suprailiac, subscapular, middle thigh and leg)	In the absence of predictive equations, apply a summary of folds
	Body perimeters (arm, waist, hip, gluteus, middle thigh, leg)	Waist circumference Waist/hip index
DEXA (in all follow-up visit)	Body composition: total fat mass, fat mass distribution	Android/gynoid fat mass ratio
Indirect calorimetry (every 2 years)	Value of resting energy expenditure Value of the respiratory coefficient	Compare with normal range
Dietary records (in all follow-up visit)	72 h registration Frequencies of food consumption	Assessment of energy intake, % of macronutrients and energy distribution, % of energy in each meal compared to the total Valuation of food and beverages consumption
Blood test (every years) Blood pressure (in all follow-up visit)	Fasting glycemia, insulinemia and lipidemia Leptin, Ghrelin, anorexigenic gastrointestinal hormones: Cholecystokinin (CCK), Tyrosine-tyrosine peptide (PYY), Pancreatic polypeptide (PP), Insulinotropic glucose-dependent polypeptide (GIP), Glucagon-like petptide 1 (GLP-1), Oxintomodulin (OXM), Glucagon-like petptide 2 (GLP-2), orexigenic and anorexigenic neuropeptides (Corticotropin-releasing hormone (CRH), melanocortin, agouti protein, cocaine- and amphetamine-regulated transcript (CART) and Melanin-concentrating hormone (MCH)) Cortisol, noradrenalin, thyroid hormones	Compare with normal range

During the growing period, current protocols suggest annual follow-up visits. In adults, the frequency of visits can be every 2 years. Follow-up visits throughout childhood and adolescence are aimed at detecting changes that could induce a risk situation that can be treated immediately. For instance, the early development of obesity could be detected triggering the implementation of a multidisciplinary protocol with the patient’s agreement to ensure motivation and compliance. Food education must be intensified, along with psychological therapy and a reinforced physical activity plan. It could be also necessary to perform analytical evaluation of patients to study glucose metabolism, orexigenic hormones, anorexics and stress hormones, such as cortisol, and observe their relationship with obesity, stress and constant eating in achondroplasia.

## Conclusion

Today they are some evidences showing that, in many diseases, the quality of food could influence the development of other complications. However, today’s usage often do not consider a full nutrition protocol to be of importance in these disease management. Although it seems difficult to prevent obesity in achondroplasia patients because of the precocity of this phenomenon, it is essential to adapt the patient’s medical management. Recent scientific discoveries allow us to better understand the origin of obesity in the context of achondroplasia and the tools that can be used to characterize it. Although, more studies are necessary to better characterize this phenomenon, all of these observations have a short-term impact permitting to improve and include nutritionist in the medical management of patient with achondroplasia. The most important is that these observations highlight the importance to perform a personalized medical management for each patient from birth to adulthood in order to improve patient’s life.

## Data Availability

Not applicable.

## Abbreviations

BMI: Body mass index
CART: Cocaine- and amphetamine-regulated transcript
CCK: Cholecystokinin
CRH: Corticotropin-releasing hormone
DEXA: Dual Energy X-ray Absorptiometry
FGFR3: Fibroblast Growth Factor Receptor 3
GIP: Insulinotropic glucose-dependent polypeptide
GLP-1 (2): Glucagon-like Peptide 1 (2)
MAPK: Mitogen Activated Protein Kinase
MCH: Melanin-concentrating hormone
OGTT: Oral Glucose Tolerance Test
OXM: Oxintomodulin
PP: Pancreatic polypeptide
PYY: Tyrosine-tyrosine peptide
QoL: Quality of Life
REE: Resting Energy Expenditure
